# The SAGA/TREX-2 subunit Sus1 binds widely to transcribed genes and affects mRNA turnover globally

**DOI:** 10.1186/s13072-018-0184-2

**Published:** 2018-03-29

**Authors:** Varinia García-Molinero, José García-Martínez, Rohit Reja, Pedro Furió-Tarí, Oreto Antúnez, Vinesh Vinayachandran, Ana Conesa, B. Franklin Pugh, José E. Pérez-Ortín, Susana Rodríguez-Navarro

**Affiliations:** 1 Gene Expression and RNA Metabolism Laboratory, Instituto de Biomedicina de Valencia, Consejo Superior de Investigaciones Científicas (CSIC), Jaime Roig 11, 46010 Valencia, Spain; 2 Gene Expression and RNA Metabolism Laboratory, Centro de Investigación Príncipe Felipe (CIPF), Eduardo Primo Yúfera 3, 46012 Valencia, Spain; 3 Departamento de Genética and E.R.I. Biotecmed, Facultad de Biología, Universitat de València, C/Dr. Moliner 50, 46100 Burjassot, Spain; 4 Department of Biochemistry and Molecular Biology, Center for Eukaryotic Gene Regulation, The Pennsylvania State University, Pennsylvania, PA 16802 USA; 5 Genomics of Gene Expression Laboratory, Centro de Investigación Príncipe Felipe (CIPF), Eduardo Primo Yúfera 3, 46012 Valencia, Spain; 6 Departamento de Bioquímica y Biología Molecular and E.R.I. Biotecmed, Facultad de Biología, Universitat de València, C/Dr. Moliner 50, 46100 Burjassot, Spain; 7 Microbiology and Cell Science Department, Institute for Food and Agricultural Sciences, University of Florida, P.O. Box 110700, Gainesville, FL 32611-0700 USA; 8 Genetics Institute, University of Florida, 2033 Mowry Road, Gainesville, FL 32610 USA; 9 Present Address: Inserm Avenir: ‘Biology of Repetitive Sequences’-Institute of Human Genetics, CNRS UPR1142, Montpellier, France; 10 Present Address: Genentech Inc., South San Francisco, CA USA

**Keywords:** Sus1, SAGA, Transcription, GRO, ChIP-exo

## Abstract

**Background:**

Eukaryotic transcription is regulated through two complexes, the general transcription factor IID (TFIID) and the coactivator Spt–Ada–Gcn5 acetyltransferase (SAGA). Recent findings confirm that both TFIID and SAGA contribute to the synthesis of nearly all transcripts and are recruited genome-wide in yeast. However, how this broad recruitment confers selectivity under specific conditions remains an open question.

**Results:**

Here we find that the SAGA/TREX-2 subunit Sus1 associates with upstream regulatory regions of many yeast genes and that heat shock drastically changes Sus1 binding. While Sus1 binding to TFIID-dominated genes is not affected by temperature, its recruitment to SAGA-dominated genes and RP genes is significantly disturbed under heat shock, with Sus1 relocated to environmental stress-responsive genes in these conditions. Moreover, in contrast to recent results showing that SAGA deubiquitinating enzyme Ubp8 is dispensable for RNA synthesis, genomic run-on experiments demonstrate that Sus1 contributes to synthesis and stability of a wide range of transcripts.

**Conclusions:**

Our study provides support for a model in which SAGA/TREX-2 factor Sus1 acts as a global transcriptional regulator in yeast but has differential activity at yeast genes as a function of their transcription rate or during stress conditions.

**Electronic supplementary material:**

The online version of this article (10.1186/s13072-018-0184-2) contains supplementary material, which is available to authorized users.

## Background

Expression of protein-coding genes is a coordinated process that consists of a series of linked steps in mRNA fate: transcription, processing, export to the cytoplasm, translation and degradation. Regulation of gene expression is essential to cellular metabolism and to adaptation to stress and developmental processes. A key step in eukaryotic transcription is the binding of a sophisticated repertoire of regulators that collectively function to control the timing of expression of each gene. Eukaryotic RNA polymerase II (RNAPII) genes have been classically divided into two main groups attending to the presence or absence of the TATA sequence in their promoters [1]. While transcription of all genes requires the loading of the TATA-binding protein (TBP) onto their promoter sequences, differences exist in the co-regulators that are used. TATA-like containing genes are mainly dependent on the general transcription factor IID (TFIID), while the SAGA coactivator (Spt–Ada–Gcn5 acetyltransferase) plays a relatively greater role in the expression of TATA-box genes [2, 3]. Accordingly, yeast genes have been called TFIID- and SAGA-dominated, depending on which complex has the strongest influence on their transcription [2]. The SAGA complex is widely conserved from yeast to humans and acts during different stages of gene expression (reviewed in [4–6]). Interestingly, and in contrast to SAGA, TFIID seems to play a role restricted to preinitiation events. In addition to SAGA, a similar coactivator, referred to as SLIK (SAGA-LIKe), has also been described [2, 3, 7–9]. The subunit composition is identical to that of SAGA, except that in SLIK the Spt7 subunit is C-terminally truncated by proteolytic cleavage, leading to release of the Spt8 subunit.

SAGA and SLIK are organized in submodules [4–6, 10] and have two inherent catalytic activities on nucleosomal histones: histone acetyltransferase activity (HAT) [11, 12] and histone H2B deubiquitylation (DUB) [13–15]. Both activities are essential for the post-translational modification of the histone tails, which provokes a shift from compact/inactive to open/active chromatin states [16]. SAGA is formed by distinct modules that work together to localize the HAT and DUB activities of the complex in the gene promoter, a necessary step for enhancing transcriptional activation, facilitating elongation and promoting nucleosome eviction.

Apart from SAGA-dependent events affecting histone modifications during transcription initiation, several studies have identified SAGA roles other than those of upstream regions. In fact, yeast SAGA subunits are not restricted to promoters, as they also localize to coding sequences of specific genes [17–19]. In these locations, SAGA subunits co-transcriptionally promote nucleosome eviction through Gcn5’s HAT activity and the deubiquitylation of H2B by Ubp8. Furthermore, SAGA interacts functionally and physically with TREX-2 (transcription and export complex 2), a nuclear pore-associated complex involved in genome stability, mRNA biogenesis and export [20–23]. Through components of the SAGA deubiquitylation module, SAGA and TREX-2 co-localize at the nuclear periphery [24]. Sus1, a shared SAGA/SLIK/TREX-2 factor, also localizes to promoters and coding regions of some SAGA-dominated genes (*ADH1* and *GAL1*), where it promotes the interaction with export factors, contributing to the coupling of transcription and mRNA metabolism [25].

Deciphering how SAGA’s different enzymatic activities control the expression of their target genes is a major challenge in fundamental transcription research. Classical results from genome-wide approaches suggested that SAGA binds to a subset of promoters in yeast [26–30] that are downregulated in SAGA mutants [2]. SAGA predominates at promoters of highly regulated stress-responsive TATA-box-containing genes, which comprise about 10% of the genome. Interestingly, there is not a clear correlation between the binding of SAGA and the misregulation of bound genes [28, 31, 32]. In fact, it has been shown that > 99% of the expressed yeast genome is positively regulated by the overlapping involvement of TFIID and SAGA [2]. Moreover, enzymatic activities of SAGA subunits, i.e. histone acetylation and deubiquitylation, seem to play roles that go beyond the transcriptional initiation of SAGA-dominated genes. For example, a previous study showed that SAGA acetylates histones at promoter regions as well as deubiquitylates histone H2B from transcribed region of all expressed RNAPII genes [33]. Furthermore, the separated roles of SAGA and TFIID in the regulation of TATA-containing and TATA-like genes have recently been reconsidered. New results confirm that transcription of nearly all genes is dependent on TFIID [34] and that SAGA may act as a general co-factor required for RNAPII transcription in *Saccharomyces cerevisiae* [35]. In contrast, other studies indicate that SAGA is not a general transcription factor in flies and mammals [36–38].

In this study, we show for the first time the genome-wide recruitment of the SAGA/TREX-2 factor, Sus1, to RNAPII-transcribed genes. Sus1 accumulates at upstream activator sequences (UASs) at a variety of gene classes: ribosomal protein (RP), SAGA-dominated and TFIID-dominated genes. The amount of Sus1 binding is correlated with the transcription rate (TR) of target genes. Analyses of Sus1 chromatin recruitment under heat shock revealed that while this stress scarcely affected Sus1 binding to TFIID-dominated genes, the binding pattern at SAGA and RP genes was substantially affected. Consistent with the genome-wide association of Sus1 to UAS, we found that transcription rates and stabilities of most yeast transcripts are changed in the absence of Sus1. Lastly, we demonstrate that both SAGA and SLIK participate in Sus1 recruitment to SAGA-dominated genes. In sum, our results support a model in which Sus1 is intimately tied to the expression of yeast RNAPII genes.

## Results

### Sus1 is recruited genome-wide to promoters of RNAPII-transcribed genes

**Fig. 1:**
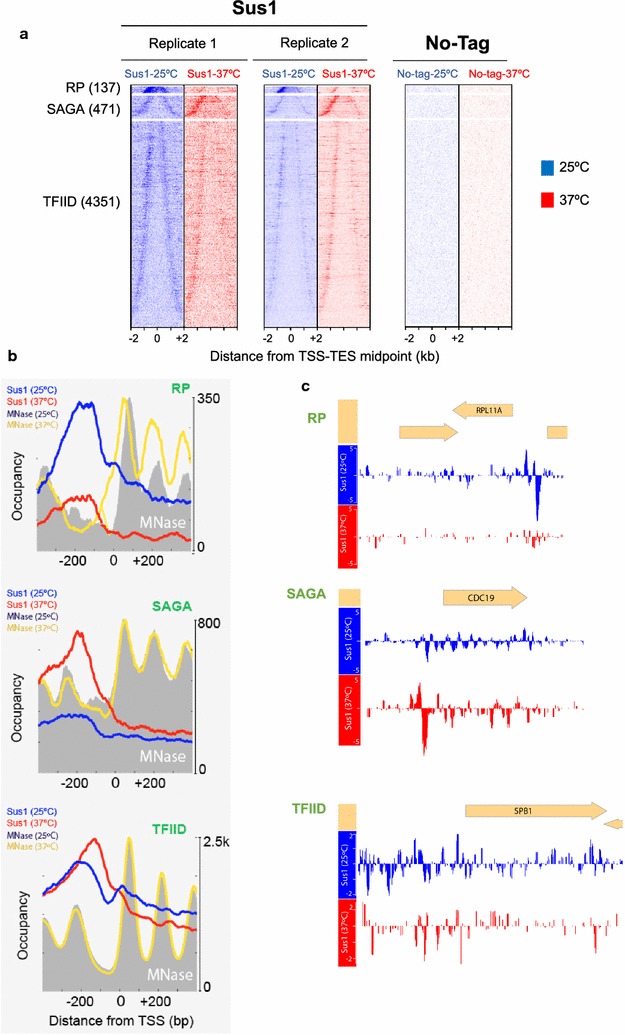
Positional organization of Sus1 before and after heat stress. **a** Heat map showing shifted 5′-end sequencing reads (tags) for Sus1-WT at 25 °C (blue) and after 15 min at 37 °C (red), aligned by the midpoint in between transcription start site (TSS) and transcription end site (TES). Data presented are divided into three subgroups: ribosomal protein genes (RP, *n* = 137), SAGA-dominated genes (SAGA, *n* = 471) and TFIID-dominated genes (TFIID, *n* = 4351) genes and sorted by gene length in each subgroup. The results of two replicates are shown. As a control, the signal of an isogenic strain bearing no-tagged Sus1 was also monitored (No-tag). **b** Gene-averaged 5′-ends of shifted relative read counts (representing points of cross-linking) of Sus1-WT at 25 °C (blue line) and at 37 °C (red line) around the transcription start site (TSS) in three gene classes: ribosomal protein (RP) genes, SAGA-dominated genes and TFIID-dominated genes, with TSS oriented to the right. Nucleosomes (based on MNase ChIP-seq) are plotted and based on values from [39]. Abrupt heat shock at 37 °C (yellow line) and 25 °C (grey fill) is shown. The resulting normalized ratios were plotted. Note that ordinate scales vary for the three gene classes due to differences in the number of genes in each class. c) Signal tracks, showing unshifted ChIP-exo tag 5′-end reads for Sus1-wt at 25 °C and 37 °C at RP-dependent gene *RPL11* (upper panel), at SAGA-dependent gene *CDC19* (middle panel) and at a TFIID-dominated gene *SPB1* (lower panel) are shown

To identify the target genes of Sus1 on the genome, we analysed the genome-wide chromatin recruitment of Sus1 by chromatin immunoprecipitation-exonuclease (ChIP-exo) experiments. Heat maps showing shifted 5′-end sequencing reads (tags) for Sus1-WT at 25 °C (blue) and after 15 min at 37 °C (red) were aligned by the midpoint between transcription start site (TSS) and transcription end site (TES) (Fig. 1a). To gain insights into possible Sus1-binding specificities, we examined the Sus1 chromatin association separately for groups of genes with different promoter organizations. Yeast genes were divided into three subgroups: ribosomal protein (RP), SAGA-dominated (SAGA) and TFIID-dominated (TFIID) genes [2] and sorted by gene length in each subgroup (Fig. 1a). The bell-shaped profiles of Sus1 binding to SAGA and TFIID classes suggested that at 25 °C, Sus1 binds near both 5′ and 3′ gene ends, whereas in RP genes the association of Sus1 corresponds mainly to 5′ end (Fig. 1a). To better study Sus1-binding pattern, we joined in a metagene representation the 5′-end profiles of shifted sequencing reads—representing points of cross-linking from Sus1-WT at 25 °C (blue) and the nucleosome (MNase) traces at 25 °C (grey fill) taken from [39] (Fig. 1b). Both profiles were plotted around the transcription start site (TSS) oriented to the right. Specific Sus1 binding to RP, SAGA and TFIID genes is depicted in three independent panels. Generally, we found that Sus1 binds preferentially to the UAS which overlaps with nucleosome-free regions (NFRs) of all these three gene classes. Under standard conditions, most Sus1 binding overlaps the − 1 nucleosome at position ~ − 200 and part of the NFR in all cases. These results were validated by ChIP-qPCR on a subset of TFIID genes using a different tagged version of Sus1 (Additional file 1: Fig. S1).

### Sus1 association to UAS depends on growth conditions

Distinct mobilization of SAGA- and TFIID-linked regulators occurs during acute heat shock [28, 30]. The heat-shock response in yeast involves transient changes in transcriptional rate, mRNA levels and mRNA stabilities of approximately 1000 genes within 15 min of the stress [40–42]. Different gene clusters showed accumulation of different complexes that regulate transcription, and SAGA-dominated genes had the largest increase in occupancy of many regulators [28]. To study the extent to which the genome-wide association of Sus1 to all gene classes is affected by heat shock, we conducted ChIP-exo experiments of Sus1 binding at 37 °C (Fig. 1a, red line) and compared to data at 25 °C (Fig. 1a, blue line). We discovered that while heat shock barely affected Sus1 binding to TFIID genes, it significantly shifted the Sus1-binding pattern at SAGA and RP genes. While Sus1 binding to RP genes was significantly reduced, the presence of Sus1 at SAGA-dominated genes was augmented. Examples of Sus1 association to each class of genes at both temperatures are shown in Fig. 1c. Gene set enrichment analysis (GSEA) of the Gene Ontology (GO) terms of the top in the ordered list of bound genes at each temperature indicated that at 25 °C Sus1 preferentially binds to *cytoplasmic translation* and *ribosomal subunit* genes, while at 37 °C *protein folding* and *response to heat* genes are most associated with Sus1 (Additional file 2: Fig. S2).

### Sus1 affects the synthesis and stability of mRNAs at a genome-wide level

**Fig. 2:**
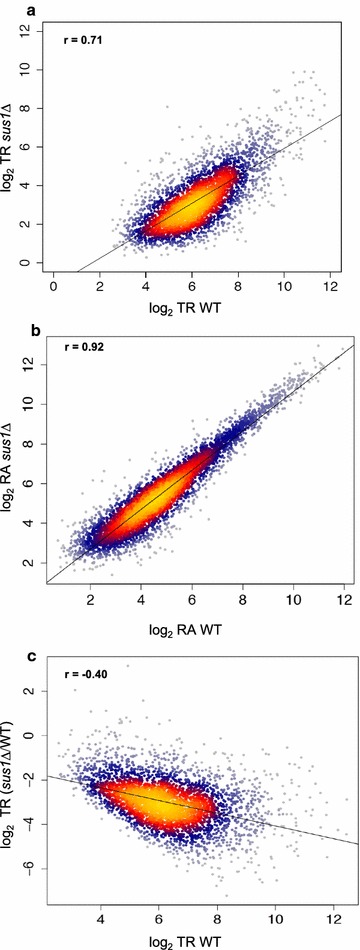
Genomic effects of Sus1 depletion on mRNA turnover. **a** Plot of the transcription rates (TR) of 3757 yeast genes in the *sus1Δ* deletion mutant versus the wild-type strain. **b** Plot of the mRNA levels (RA) of 5216 yeast genes in the *sus1Δ* deletion mutant versus the wild-type strain. **c** Plot of the fold change of TR of 3757 yeast genes in the *sus1Δ* mutant against the TR level of the WT. Note that all graphs are in log_2_ scales of arbitrary units. Pearson R of the cloud to a linear fitting is shown

In the light of our ChIP-exo results, we decided to re-investigate the genome-wide role of Sus1 in transcription. In a previous study, we showed that the deletion of *SUS1* affects the steady-state levels of around 10% of mRNAs [20]. In that work, total RNA was obtained from *sus1Δ* and wild-type (WT) cells and gene expression levels were analysed using DNA arrays. However, as the final amount of any transcript is determined by the balance between its transcription and degradation rates [43], we conducted new genomic run-on (GRO) experiments [44] and obtained the nascent transcription rate (TR) for each transcript in WT and *sus1Δ* cells (see details at Materials and Methods). In addition, we used previously published relative mRNA abundance (RA) estimates [20] for comparative purposes. We found that the TRs of *sus1Δ* and WT cells were much less correlated (*R* = 0.71; Fig. 2a) than their RAs (*R* = 0.92; Fig. 2b). To evaluate how the absence of Sus1 affects the TR, we represented the mutant versus WT ratio of TR values against TR in the WT. This analysis showed that the absence of Sus1 provokes a genome-wide decrease in TR (most data points are below the ratio 1) and that this effect increases with the absolute TR (Fig. 2c). GSEA of genes ranked by their TR ratios (*sus1*Δ/WT) revealed that *translation*-related genes tend to have high TR ratios, while *energy derivation* genes are at the lowest TR ratios (Additional file 3: Fig. S3).

**Fig. 3:**
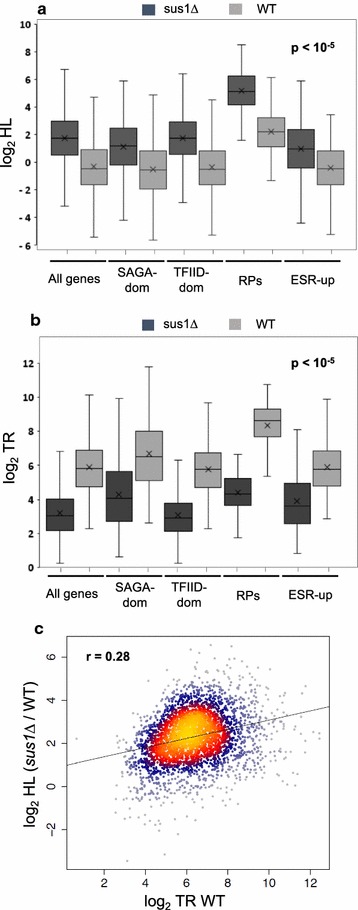
Genomic effects of Sus1 depletion on changes in mRNA half-lives. **a** Box-and-whisker plot of the changes in mRNA stability half-lives (HL) in *sus1Δ* mutant (dark grey) and the wild-type strain (light grey). All comparisons show a statistically significant HL increase of *sus1Δ* in relation to wild-type strains (Wilcoxon signed-rank test *p* value < 10^−5^). Note that SAGA- and TFIID-dominated genes have similar average increase in HLs (3.3- vs. 4.73-fold) but that ribosomal protein-coding genes (RP) are much more increased in HLs (7.7-fold) than environmental stress response activated (ESR-up) genes (2.75-fold). **b** Box-and-whisker plot of the changes in TR in the *sus1Δ* mutant (dark grey) and the wild-type strain (light grey). All comparisons show a statistically significant TR decrease of *sus1Δ* in relation to wild-type strains (Wilcoxon signed-rank test *p* value < 10^−5^). Note that the decrease in RP genes is much higher (19.4-fold) than in SAGA (5.5-fold), TFIID (7.1-fold) or ESR-up (4.4-fold) genes. **c** Plot of the fold change in HL in the *sus1Δ* mutant against the TR level of each gene in the WT strain. Note that both axes are in log_2_ scales of arbitrary units. Pearson *R* of the cloud to a linear fitting is shown

Lower TRs can be compensated by global mRNA stabilization, giving rise to similar RAs [43]. To understand the role of Sus1 on mRNA stability, the distribution of RA/TR ratios (a proxy of mRNA half-lives, HL) between the mutant and WT strains was compared. We found that, globally, *SUS1* deletion increased global mRNA stability (Fig. 3a, all genes), but the magnitude of the effect was different for distinct gene classes. While SAGA- and TFIID-dominated genes increased their HL ratio by 3.3 and 4.73 times, respectively, in the *sus1*Δ mutant, the effect in RP genes was higher (7.7 times), while environmental stress-response upregulated (ESR-up) [40] transcripts behaved similarly to SAGA- and TFIID-dominated genes (Fig. 3a). These results tend to compensate the differences in TR observed in absence of Sus1 for each set of genes (Fig. 3b) and suggest that Sus1 causes increased mRNA turnover when genes are transcribed more frequently. To verify this observation, we plotted fold change in HL in *sus1*Δ versus WT against TR in WT and observed a positive trend, indicating that Sus1 is more effective at destabilizing mRNA of highly expressed genes (Fig. 3c). Similarly to TR ratios, GSEA analysis of HL *sus1*Δ/WT ratios found a statistically significant over-representation of translation-related GO categories within the most stabilized mRNAs, and reciprocally, of energy derivation-related GOs within the less-stabilized ones (Additional file 4: Fig. S4). We conclude that Sus1 destabilizes RNAPII-transcribed mRNAs, with its impact being generally greater the more a gene is expressed.

### Sus1 binding shows a positive correlation with transcriptional rate

**Fig. 4:**
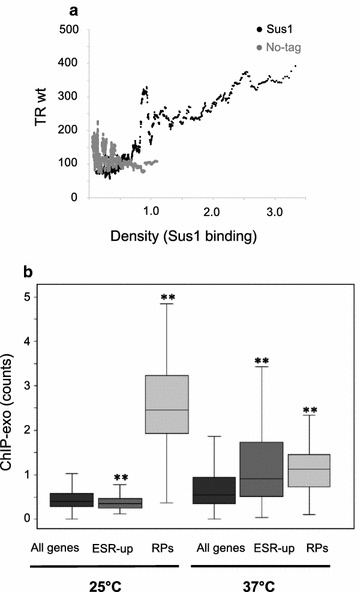
The binding of Sus1 to UAS correlates with the gene TR. **a** A sliding window plot of the transcription rate (TR) in arbitrary units versus the total reads counted in each gene in the Sus1 (black dots) or the no-tag (grey dots) of the ChIP-exo experiments. A window of 100 genes was used in both cases. **b** Box-and-whisker plot of the Sus1 binding to yeast genes. Note that the binding to ESR-up genes is lower than the global genome average in a wild-type strain and increases with temperature, whereas the opposite trend is observed for RP genes. Differences between ESR-up or RP genes and all genes are statistically significant for both temperatures (Wilcoxon signed-rank test *p* value < 10^−5^)

Since our results showed a positive relationship between HL fold change and TR levels affected by Sus1 deletion (Fig. 3c), we next investigated to which extent Sus1 binding correlates with transcriptional activity by plotting the number of Sus1 ChIP-exo reads at UAS versus TR values obtained from our GRO experiment through a sliding window representation. We found a positive trend of increasing Sus1 binding with the increase in TR for all genes (Fig. 4a, black dots), that was not observed for the no-tag ChIP-exo data (Fig. 4a, grey dots).

Our analyses revealed that upon heat shock Sus1 binding to RP genes was significantly reduced, while its presence at SAGA-dominated genes was augmented (Fig. 1b). It has been shown that many SAGA-dominated genes are part of the ESR-up transcripts [2, 40, 45–48], whereas RP genes are a fundamental part of the ESR-down response. Therefore, we hypothesized that Sus1 would release from RP genes and associate preferentially to ESR-up genes upon stress [30]. To confirm this hypothesis, the binding of Sus1 to RP and ESR-up genes was studied before and after this temperature shift. As shown in Fig. 4b, Sus1 binding to ESR-up genes was lower than the global genome average in a wild-type strain at 25 °C. In contrast, association of Sus1 to ESR-up genes was significantly augmented upon heat shock. In contrast, Sus1 binding to RP genes dropped abruptly when cells were incubated at 37 °C for 15 min (Fig. 4b). These results support an active role for Sus1 in the transcriptional regulation of the environmental stress response in yeast.

### SLIK participates in Sus1 recruitment to SAGA-dominated genes

**Fig. 5:**
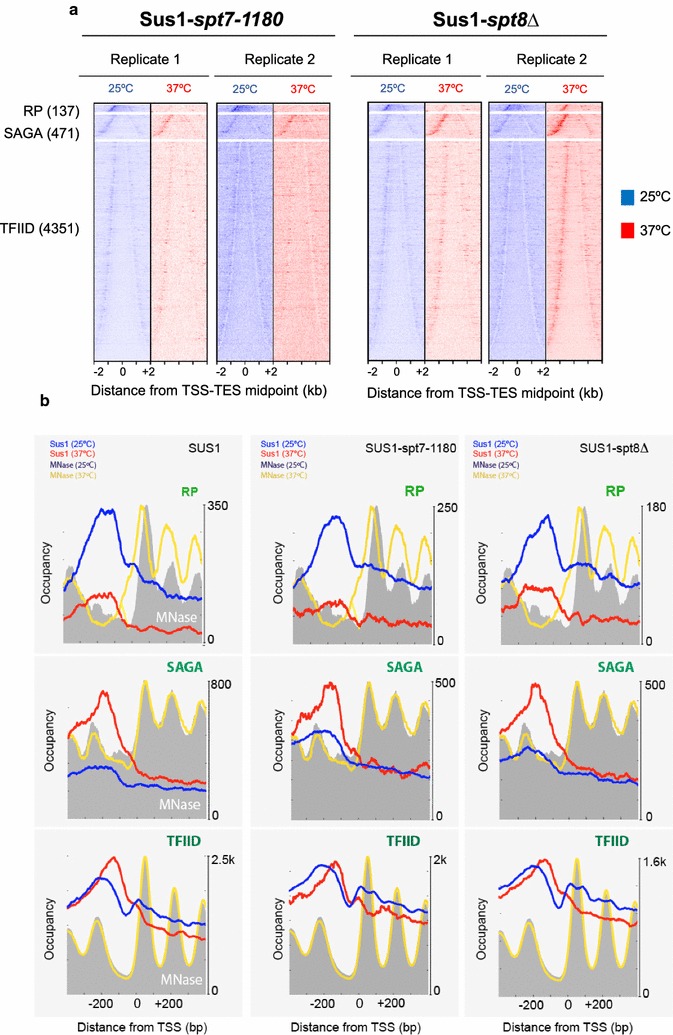
Positional organization of Sus1 in SLIK-constitutive mutants. **a** Heat map showing shifted 5′-end sequencing reads (tags) for Sus1 in *spt71180* (left panel) and *spt8Δ* (right panel) at 25 °C (blue) and after 15 min at 37 °C (red), aligned by the midpoint in between the transcription start site (TSS) and transcription end site (TES), split into three subgroups: RP, SAGA and TFIID genes and sorted by gene length in each subgroup. The results of two replicates are shown. **b** Gene-averaged 5′-ends of shifted relative read counts (representing points of cross-linking) of Sus1-WT (left panel), Sus1 in *spt71180* (middle panel) and *spt8Δ* (right panel) at 25 °C (blue) and after 15 min at 37 °C (red) around the transcription start site (TSS) in three gene classes: ribosomal protein (RP) genes, SAGA-dominated genes and TFIID-dominated genes, with TSS oriented to the right. Nucleosomes (based on MNase ChIP-seq) are plotted and based on values from [39]. Abrupt heat shock at 37 °C (yellow line) and 25 °C (grey fill) is shown. The resulting normalized ratios were plotted. Note that ordinate scales vary for the three gene classes due to differences in the number of genes in each class

We previously addressed the contribution of SAGA and TREX-2 to Sus1 chromatin association via investigating Sus1 recruitment to *ARG1* in *ubp8Δ* (SAGA) and in *sac3Δ* (TREX-2) mutants [18]. The experiments showed that Sus1 binding to both complexes was required for correct recruitment to the *ARG1* UAS [18]. Sus1 is also part of SLIK [49]. The functional differences between SAGA and SLIK are not fully understood, and some studies have proposed that they could play different roles at promoter or coding sequences [8]. Since our results highlighted a global chromatin association pattern for Sus1, we wondered whether this remains true for Sus1 as part of SLIK. To this end, we analysed the ChIP-exo genome-wide binding profile of Sus1 in *spt8Δ* and Spt7 C-terminal-truncated mutant (*spt7*-*1180*) in which SLIK, but not SAGA, exists [9, 49, 50], at 25 °C and at 37 °C. Heat maps showing Sus1 in *spt7*-*1180* and *spt8Δ* strains at 25 °C (blue) and after 15 min at 37 °C (red) were aligned to RP, SAGA-dominated and TFIID-dominated gene classes. Similar to the results observed for Sus1 in a WT background (Fig. 1a), Sus1 in *spt8Δ* and Spt7 C-terminal-truncated mutant (*spt7*-*1180*) bound to both 5′ and 3′ gene ends, whereas at RP genes the association corresponded mainly to the 5′-end (Fig. 5a). Figure 5b shows the metagene 5′-ends profiles of shifted sequencing reads of Sus1-WT, Sus1 in *spt7*-*1180* and *spt8Δ* strains at 25 °C (blue line) and upon heat shock (red line). Data are plotted around the transcription start site (TSS) oriented to the right, together with nucleosome (MNase) traces at 25 °C (grey fill) and after heat shock (yellow trace) from [39]. Sus1 recruitment is slightly diminished at SAGA-dominated genes when only SLIK is present, indicating that both SAGA and SLIK participate in Sus1 recruitment to SAGA-dominated genes.

## Discussion

In this study, we demonstrate a general role for Sus1 in transcription and mRNA stability in yeast cells. Sus1 is recruited to the upstream activation sequences of many genes and is necessary for mRNA synthesis of a large number of transcripts independently of the presence of a TATA sequence. The classical model of the role of SAGA/TFIID in regulating transcription states that they possess distinct but overlapping selectivity for promoters, thereby fostering a cascade of events that leads to the correct expression of their targeted genes. In classical studies, TATA-containing genes were relatively more dependent on SAGA, whereas TATA-like genes were relatively more dependent on the general transcription factor TFIID [2]. Numerous ChIP studies of SAGA factors revealed a picture of confined recruitment of SAGA to specific promoters and to some coding sequences [17–19, 28, 30, 51]. However, recent work that tried to delimit the specificity of SAGA regulation concluded that TFIID and SAGA are more equivalent than initially thought [34, 35]. These studies reported that SAGA-associated enzymatic activities act more widely at all transcribed genes [33]. In particular, it was shown that histone acetylation and deubiquitylation mediated by the SAGA-associated proteins Gcn5 and Ubp8, respectively, occur genome-wide, suggesting that SAGA has a broader role in gene expression. However, we suggest that contributions from subcomplexes that are not part of SAGA could not be excluded. The current view is further supported by two recent publications describing TFIID and SAGA as general cofactors for RNAPII transcription in yeast [34, 35]. SAGA factors Spt7, Spt3, Spt8 and Ubp8 were reported to associate with UASs at a majority of genes, which contrasts with recent results showing much more restricted localization [30]. Sus1 (this study) and SAGA ([30]) recruitments are enriched for highly transcribed genes and peak in the -150/-200-bp region coinciding with the UAS and NFR of many yeast genes, with particular enrichment of SAGA subunits at RP and SAGA-dominated genes. Further work is needed to resolve the discrepancy between the discordant studies.

Seminal studies that established the regulatory role of SAGA and the functional clustering of its subunits measured steady-state RNA levels, without differentiating between transcription and degradation rates [2, 31]. In this work, we measured separately transcriptional rate and mRNA abundance, and discovered that i) the absence of *SUS1* has a strong negative effect on the transcription rate and ii) mRNAs were generally more stable in the *sus1Δ* mutant. Similar phenotypes have recently been described for the SAGA/SLIK structural mutants *spt7Δ* and *spt20Δ* [33] and for some other SAGA subunits [35]. The GRO analyses of *sus1Δ* presented here complement these observations, suggesting that Sus1 is part of the SAGA-related factors that influence the stability of RNAPII-transcribed mRNAs. However, since Sus1 is a shared component of SAGA/SLIK and TREX-2, we cannot attribute unambiguously the observed Sus1-dependent effect on transcription only to SAGA/SLIK. Further work is required to study the contribution of TREX-2 to this phenotype.

We observe that genes more stabilized by absence of *SUS1* tend to have higher TR. This could reflect a compensatory crosstalk mechanism in which the general reduction in TR by *SUS1* deletion is compensated by increase in HL in order to maintain mRNA levels, or vice versa. Such effect has previously been reported for decay factors [52, 53] and for transcription factors [54, 55]. In this way, Sus1 would participate in the co-transcriptional imprinting on mRNA molecules that regulates transcript fate [52, 56]. The fact that Sus1 is also involved in mRNA export, which is known to be coupled to upstream events [57], makes it an excellent candidate to participate in the mRNA imprinting of different classes of transcripts. Although the stability of all types of mRNAs (SAGA, TFIID and RP) depends on Sus1, we observed differences among them. On average, Sus1 absence affects more the HL of RPs mRNAs than TFIID-dominated and SAGA-dominated ones. RP and TFIID-dominated genes require a higher increase in HL to buffer for a global mRNA homoeostasis which is expected under standard conditions. Sus1 association to TFIID-dominated genes is barely affected by heat, probably because relative few TFIID genes are heat-shock-induced. In contrast, upon stress Sus1 relocates preferentially to promoters of SAGA-dominated genes. This change is maximal for promoters that are part of the environmental stress response (ESR-up). Since the RP genes are a fundamental part of the ESR-down response, a reallocation of Sus1 from RP genes to ESR-up genes as part of the heat-shock ESR is expected. This behaviour is consistent with the observation that Sus1 associates with elongating RNAPII [18], which is reallocated from RP to ESR-up genes upon stress. One possibility is that this phenotype is imposed by the chromatin organization of each gene class, being ESR-up genes controlled by SAGA to allow fine-tuned transcriptional changes. We speculate that SAGA-dependent histone modifications might become more crucial in these chromatin contexts than in genes that require less expression plasticity (for instance, constitutive TFIID genes). Additionally, data indicate that SAGA and TFIID have different functional spectra, and while TFIID is exclusively involved in PIC assembly, SAGA subunits also participate in transcript elongation [33]. Consequently, the SAGA pathway provides a greater dynamic range of mRNA output and more opportunities for transcriptional regulation [28].

SAGA and SLIK share all but two of their components: Spt8 and full Spt7. The distinctive roles of the two complexes are still not clear and are difficult to address because of this high degree of overlap. We examine here an “ONLY-SLIK” phenotype by using mutants of *spt8Δ* and truncated *spt7*-*1180*. We addressed the contribution of SLIK to Sus1 recruitment by demonstrating that SLIK contributes to recruitment of Sus1 genome-wide, along with SAGA. Therefore, these results provide insights into the shared roles of SAGA and SLIK.

## Conclusions

We propose here that under standard conditions Sus1 is recruited at a basal level to the UAS regions of expressed RP, SAGA-dominated and TFIID-dominated genes, at levels that reflect their transcriptional activity. Upon heat stress, Sus1 is preferentially associates with promoters of SAGA-dominated genes (especially ESR-up genes) in parallel with gene activation. Since Sus1-dependent deubiquitylation is genome-wide [58], Sus1 could facilitate the “fast” binding of the SAGA-DUB module in all these genomic locations, supporting a transient interaction between at least some SAGA/SLIK subunits and coding regions. Furthermore, since Sus1 is part of TREX-2 whose presence is enriched towards 3′ ends [23], Sus1 could contact TREX-2 to ensure a correct coupling between the different steps of the gene expression process. This is consistent with our detection of Sus1 also at the 3′ end of genes. Last, a common feature of SAGA and TFIID is the presence of a shared set of TAFs [59]. A provocative hypothesis is that a preassembled core of these shared TAFs serves as a scaffold for assembly or switching between TFIID, SAGA or SLIK complexes at yeast genes promoters.

## Methods

### Yeast strains, recombinant DNA work and microbiological techniques

The yeast strains used in this study are listed in Additional file 5: Table S1. Chromosomal integration of TAP (*URA3* marker) and MYC (*HIS3* marker) as C-terminal tags was performed as previously described [60, 61]. For gene disruption, the indicated gene was deleted by high-efficiency transformation using a PCR product amplified from the plasmid pRS400-KanMX4. All deletions and genomically tagged strains were confirmed by PCR and/or western blot analysis. Strains were grown under standard conditions.

### Chromatin immunoprecipitation (ChIP)

ChIP was performed as described [62] with minor modifications. Fifty millilitres of exponential-phase cultures grown in YPD at 25 °C was cross-linked with 1% of formaldehyde solution (Sigma) for 20 min at room temperature. The reaction was quenched with 125 mM glycine. Cells were collected and washed with Tris–saline buffer. Cells were broken in lysis buffer with glass beads, and cell extracts were sonicated in a Bioruptor (Diagenode). Ten microlitres of extract was reserved as the input, and the rest was incubated with Dynabeads^®^ Pan Mouse IgG for 2 h. Immunoprecipitates (IP) were washed, and samples were eluted at 65 °C for 20 min with 100 µL of elution buffer. Inputs and immunoprecipitation samples were incubated at 65 °C overnight to reverse the cross-linking. Samples were treated with proteinase K, and DNA was purified with phenol/chloroform/isoamyl alcohol extraction and ethanol precipitation. The list of primers for ChIP analysis and RTqPCR can be found in the Additional file 6: Table S2).

### ChIP-exo and data analyses

Two biological replicates of each ChIP-exo of Sus1-MYC-tagged protein and a no-tag strain were performed. Cells were grown in YPD at 25 °C. The experiments were performed as in [30]. The heat-shock treatment was performed at 37 °C for 15 min. Prechilled formaldehyde was added to achieve a final concentration of 1%. Reactions were quenched and cells collected by centrifugation. Solubilized sonicated chromatin was prepared from cell extracts to yield a DNA fragment size of < 500 bp. For mapping nucleosomes, data from [39] were used. DNA was trimmed in the 5–3 direction with lambda exonuclease, until stopped by formaldehyde cross-link. The resulting DNA was subjected to deep sequencing. Sequencing reads were mapped to yeast genome (sacCer3) using BWA (version 0.5.9-r16). For all figures, reads were shifted in the 3′ direction by 6 bp and strand information was removed to better reflect the point of cross-linking. To compare mock and heat-shock data sets, data were normalized to have equal number of reads. The set of SAGA-/TFIID-dominated genes were obtained from [2] (Additional file 7).

### Genomic run-on

Genomic run-on (GRO) for WT and *sus1Δ* was done and analysed as described in [44]. This method allows for the determination on nascent transcription rates genome-wide because it labels nascent mRNAs in elongating RNA polymerases. Values were transformed into true mRNA synthesis rates (TR) by dividing by the cell volume (see [43] for a detailed explanation). Aliquots of the same cell samples were used to determine mRNA levels for all yeast genes as described in [40]. Total mRNA concentration in WT and *sus1Δ* cells was determined [40] and used for normalization of mRNA levels (RA). In this way, mRNA half-lives (HL) for all mRNAs were calculated, assuming steady-state conditions, as the ratio between RAs levels and TRs.

## Additional files

**Additional file 1: Fig. S1.** Sus1 occupancy at TFIID-dependent genes was monitored by ChIP analysis of Sus1-TAP in a wild-type strain (Sus1-TAP). As a control, the signal of an isogenic strain bearing no-tagged Sus1 was monitored (No-tag). The occupancy level was calculated as the signal ratio of IP samples in relation to the input signal and relative to an internal control. The resulting normalized ratios were plotted. Error bars represent the SD from at least three independent experiments. Differences in means were assessed by Student’s independent-samples t test. P values < 0.05, indicated with an asterisk, were considered to be statistically significant.

**Additional file 2: Fig. S2.** Gene set enrichment analysis (GSEA) for the highest ChIP-exo reads. The genes were ranked according to the number of mapped reads and searched for GO terms enriched at the top of the list in comparison with the rest of the list using GSEA. The resulting list of over-represented GO terms was reduced and visualized with the ReviGO web server (http://revigo.irb.hr/). a) Binding at 25 °C. Left: Results at the Biological Process GO; right: Results at the Cellular Component GO. b) Binding at 37 °C, results are given for the Biological Process GO. The Cellular Component GO gave no results. The size of the circle for each GO term is proportional to the number of genes included, and the red colour intensity is proportional to the *p* value.

**Additional file 3: Fig. S3.** Gene set enrichment analysis (GSEA) of TR ratios (*sus1*Δ/WT). Gene Ontology (GO) terms (filtered by means of ReviGO software, see Fig, S2) over-represented at the top and at the bottom of the ranked list of TR ratio values.

**Additional file 4: Fig. S4.** Gene set enrichment analysis (GSEA) analysis of HL ratios (*sus1*Δ/WT). Gene Ontology (GO) terms (filtered by means of ReviGO software, see Fig, S2) over-represented at the top and at the bottom of the ranked list of HL ratio values.

**Additional file 5: Table S1.** Is a table listing strain used in this study.

**Additional file 6: Table S2.** Is a table listing Primers for ChIP analysis and RT-qPCR.

**Additional file 7.** ChIP-exo data analysis.

## Abbreviations

TFIID: transcription factor IID
SAGA: Spt–Ada–Gcn5 acetyltransferase
RNAPII: RNA polymerase II
SLIK: SAGA-like complex
HAT: histone acetyltransferase activity
DUB: deubiquitylation
TREX-2: transcription and export complex
RP: ribosomal protein
ESR: environmental stress response
WT: wild-type
GRO: genomic run-on
GSEA: gene set enrichment analysis
TR: transcription rate
RA: relative mRNA abundance
ChIP-exo: chromatin immunoprecipitation-exonuclease
TSS: transcription start site
TTS: transcription termination site
