# Supplementary figures and images for: The SAGA/TREX-2 subunit Sus1 binds widely to transcribed genes and affects mRNA turnover globally
Source: Epigenetics Chromatin. 2018 Mar 29;11:13. doi: 10.1186/s13072-018-0184-2 (PMC5875001; doi:10.1186/s13072-018-0184-2)

**Fig. S1**

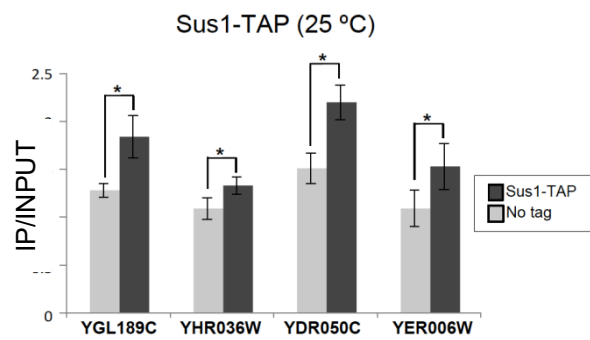

Supplement: Supplementary file 1 — Additional file 1: Fig. S1. Sus1 occupancy at TFIID-dependent genes was monitored by ChIP analysis of Sus1-TAP in a wild-type strain (Sus1-TAP). As a control, the signal of an isogenic strain bearing no-tagged Sus1 was monitored (No-tag). The occupancy level was calculated as the signal ratio of IP samples in relation to the input signal and relative to an internal control. The resulting normalized ratios were plotted. Error bars represent the SD from at least three independent experiments. Differences in means were assessed by Student’s independent-samples t test. P values < 0.05, indicated with an asterisk, were considered to be statistically significant. [file 13072_2018_184_MOESM1_ESM.pdf]

Fig. S2

a

Top Sus1 binding GO categories at 25 °C

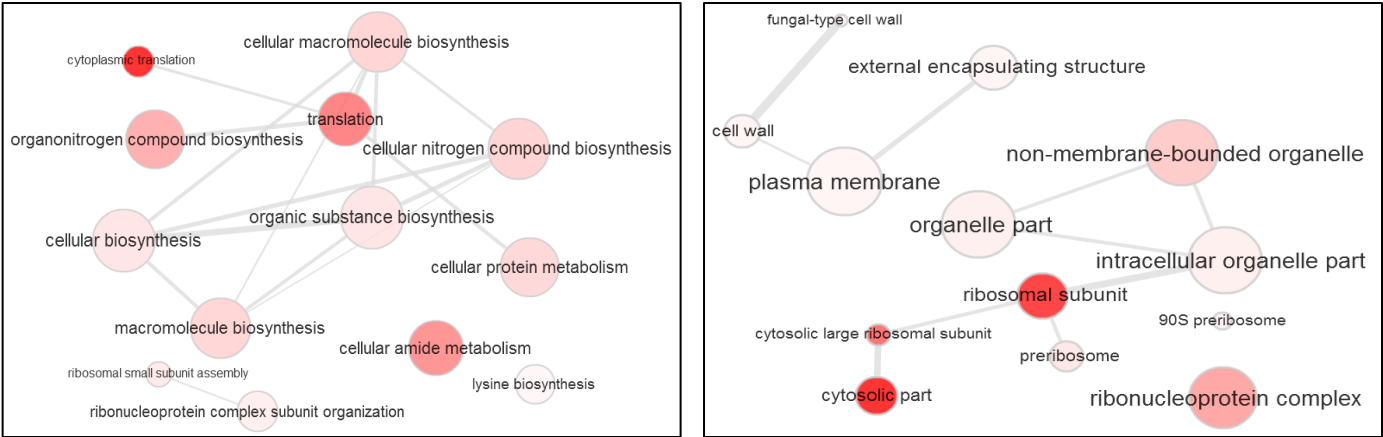

b

Top Sus1 binding GO categories at 37 °C

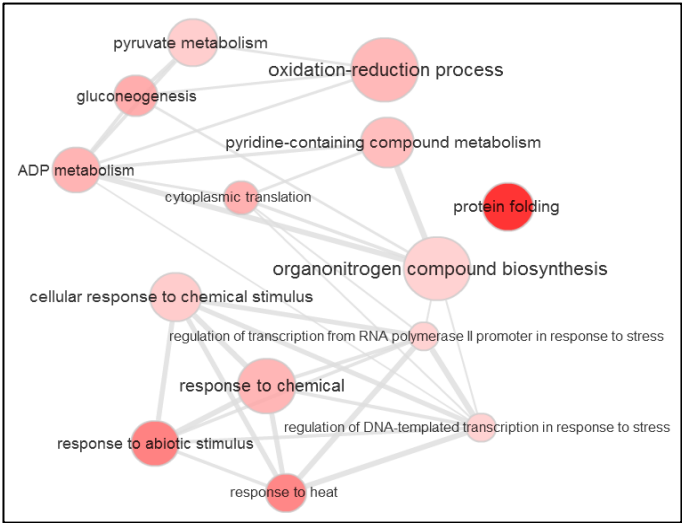

Supplement: Supplementary file 2 — Additional file 2: Fig. S2. Gene set enrichment analysis (GSEA) for the highest ChIP-exo reads. The genes were ranked according to the number of mapped reads and searched for GO terms enriched at the top of the list in comparison with the rest of the list using GSEA. The resulting list of over-represented GO terms was reduced and visualized with the ReviGO web server (http://revigo.irb.hr/). a) Binding at 25 °C. Left: Results at the Biological Process GO; right: Results at the Cellular Component GO. b) Binding at 37 °C, results are given for the Biological Process GO. The Cellular Component GO gave no results. The size of the circle for each GO term is proportional to the number of genes included, and the red colour intensity is proportional to the p value. [file 13072_2018_184_MOESM2_ESM.pdf]
