# Supplementary material for: The SAGA/TREX-2 subunit Sus1 binds widely to transcribed genes and affects mRNA turnover globally
Source: Epigenetics Chromatin. 2018 Mar 29;11:13. doi: 10.1186/s13072-018-0184-2 (PMC5875001; doi:10.1186/s13072-018-0184-2)
Supplement: Supplementary file 3 — Additional file 3: Fig. S3. Gene set enrichment analysis (GSEA) of TR ratios (sus1Δ/WT). Gene Ontology (GO) terms (filtered by means of ReviGO software, see Fig, S2) over-represented at the top and at the bottom of the ranked list of TR ratio values. [file 13072_2018_184_MOESM3_ESM.pdf]

Fig. S3

TR ratios (*sus1*Δ/WT). GO enriched terms

Top

Term ID	Description	log_{10} p-value
GO:0055114	oxidation-reduction process	-4.757
GO:0015980	energy derivation by oxidation of organic compounds	-4.281
GO:0043605	cellular amide catabolic process	-4.162
GO:0006081	cellular aldehyde metabolic process	-3.572
GO:0005978	glycogen biosynthetic process	-2.839
GO:0006091	generation of precursor metabolites and energy	-2.697
GO:0006122	mitochondrial electron transport, ubiquinol to cytochrome c	-2.423
GO:0006739	NADP metabolic process	-2.384

Bottom

Term ID	Description	log_{10} p-value
GO:0042254	ribosome biogenesis	-19.291
GO:0006412	translation	-18.636
GO:0034660	ncRNA metabolic process	-13.854
GO:0016072	rRNA metabolic process	-13.503
GO:0006396	RNA processing	-9.815
GO:0016070	RNA metabolic process	-8.083
GO:0006913	nucleocytoplasmic transport	-7.443
GO:0051169	nuclear transport	-7.443
GO:0071166	ribonucleoprotein complex localization	-6.654
GO:0033750	ribosome localization	-6.382
GO:0000466	maturation of 5.8S rRNA from tricistronic rRNA transcript (SSU-rRNA, 5.8S rRNA, LSU-rRNA)	-5.587
GO:0090501	RNA phosphodiester bond hydrolysis	-5.349
GO:0000469	cleavage involved in rRNA processing	-5.349
GO:0000478	endonucleolytic cleavage involved in rRNA processing	-4.719
GO:0044085	cellular component biogenesis	-4.684
GO:0015931	nucleobase-containing compound transport	-4.151
GO:0051640	organelle localization	-4.086
GO:0051028	mRNA transport	-4.031
GO:0006403	RNA localization	-3.975
GO:0016482	cytosolic transport	-3.336
