# Supplementary material for: The SAGA/TREX-2 subunit Sus1 binds widely to transcribed genes and affects mRNA turnover globally
Source: Epigenetics Chromatin. 2018 Mar 29;11:13. doi: 10.1186/s13072-018-0184-2 (PMC5875001; doi:10.1186/s13072-018-0184-2)
Supplement: Supplementary file 4 — Additional file 4: Fig. S4. Gene set enrichment analysis (GSEA) analysis of HL ratios (sus1Δ/WT). Gene Ontology (GO) terms (filtered by means of ReviGO software, see Fig, S2) over-represented at the top and at the bottom of the ranked list of HL ratio values. [file 13072_2018_184_MOESM4_ESM.pdf]

Fig. S4

HL ratios (*sus1Δ*/WT). GO enriched terms

Top

Term ID	Description	log_{10} p-value
GO:0042254	ribosome biogenesis	-17.519
GO:0016072	rRNA metabolic process	-14.010
GO:0006396	RNA processing	-11.614
GO:0034660	ncRNA metabolic process	-11.545
GO:0006412	translation	-9.165
GO:0016070	RNA metabolic process	-7.027
GO:0071166	ribonucleoprotein complex localization	-5.996
GO:0000466	maturation of 5.8S rRNA from tricistronic rRNA transcript (SSU-rRNA, 5.8S rRNA, LSU-rRNA)	-5.851
GO:0090501	RNA phosphodiester bond hydrolysis	-5.836
GO:0033750	ribosome localization	-5.680
GO:0006913	nucleocytoplasmic transport	-5.457
GO:0051169	nuclear transport	-5.457
GO:0000478	endonucleolytic cleavage involved in rRNA processing	-4.839
GO:0044085	cellular component biogenesis	-4.599
GO:0000967	rRNA 5'-end processing	-3.807
GO:0007049	cell cycle	-3.635
GO:0031023	microtubule organizing center organization	-3.585
GO:0051300	spindle pole body organization	-3.585

Bottom

Term ID	Description	log_{10} p-value
GO:0055114	oxidation-reduction process	-5.060
GO:0046496	nicotinamide nucleotide metabolic process	-4.788
GO:0072524	pyridine-containing compound metabolic process	-4.350
GO:0051186	cofactor metabolic process	-4.150
GO:0005978	glycogen biosynthetic process	-4.104
GO:0006740	NADPH regeneration	-4.080
GO:0019321	pentose metabolic process	-3.747
GO:0015980	energy derivation by oxidation of organic compounds	-3.638
GO:0016052	carbohydrate catabolic process	-3.431
GO:0005975	carbohydrate metabolic process	-3.179
GO:0006091	generation of precursor metabolites and energy	-3.130
GO:0044723	single-organism carbohydrate metabolic process	-2.996
GO:0016051	carbohydrate biosynthetic process	-2.987
GO:0006116	NADH oxidation	-2.917
GO:0044281	small molecule metabolic process	-2.879
GO:0006508	proteolysis	-2.796
GO:0006528	asparagine metabolic process	-2.629
GO:0009056	catabolic process	-2.594
