# Supplementary material for: The SAGA/TREX-2 subunit Sus1 binds widely to transcribed genes and affects mRNA turnover globally
Source: Epigenetics Chromatin. 2018 Mar 29;11:13. doi: 10.1186/s13072-018-0184-2 (PMC5875001; doi:10.1186/s13072-018-0184-2)
Supplement: Supplementary file 5 — Additional file 5: Table S1. Is a table listing strain used in this study. [file 13072_2018_184_MOESM5_ESM.pdf]

Supplemental Table S1. Strains used in this study.

Yeast strain	Genotype	Genotype reference
BY4741	*Mat a, leu2-Δ0, his3-Δ1, met15-Δ0, ura3-Δ0*	Euroscarf
sus1Δ	*Mat a, leu2-Δ0, his3-Δ1, met15-Δ0, ura3-Δ0 sus1::KANMX4*	This study
SUS1-TAP	*Mat a, leu2-Δ0, his3-Δ1, met15-Δ0, SUS1-TAP::URA3*	This study
SUS1-MYC	Mat a, leu2-Δ0, his3-Δ1, met15-Δ0, ura3-Δ0, SUS1-MYC::HIS3	Pascual-García et al. (2008)
SUS1-MYC *spt8Δ*	Mat a, leu2-Δ0, his3-Δ1, met15-Δ0, ura3-Δ0, *SUS1-MYC::HIS3*, *spt8::KanMX4*	This study
SUS1-MYC *spt7-1180*	Mat a, leu2-Δ0, his3-Δ1, met15-Δ0, ura3-Δ0, *SUS1-MYC::HIS3*, *spt7-1180::KanMX4*	This study
