# Supplementary material for: The SAGA/TREX-2 subunit Sus1 binds widely to transcribed genes and affects mRNA turnover globally
Source: Epigenetics Chromatin. 2018 Mar 29;11:13. doi: 10.1186/s13072-018-0184-2 (PMC5875001; doi:10.1186/s13072-018-0184-2)
Supplement: Supplementary file 6 — Additional file 6: Table S2. Is a table listing Primers for ChIP analysis and RT-qPCR. [file 13072_2018_184_MOESM6_ESM.pdf]

Supplemental Table S2. Primers for ChIP analysis and RT-qPCR.

Amplified region	Oligo name	Oligo sequence
Intergenic region (chromosome V)	*IntV*-UP	*TGTTCCTTTAAGAGGTGATGGTGA*
	IntV-DW	*GTGCGCAGTACTTGTGAAAACC*
ACT1	*ACT1*-UP	GTTTTGGATTCCGGTGATGG
	ACT1-DW	AATCTCTACCGGCCAAATCG
YGL189C ORF	YGL189C-UP	CGCTTTGCCAAAGACTTACAAC
	YGL189C-DW	CTTCTTGGCGGCATCAGCA
YHR036W ORF	YHR036W-UP	CGTTGACGAGGAGTTAGGAGG
	YHR036W-DW	CCTCATCAGCTTTAGCAGCAC
YDR050C ORF	YDR050C-UP	GTCAAGGTGTCGGTGTCATC
	YDR050C-DW	GACTGGTTCGTAAGCGACAAC
YER006W ORF	YER006W-UP	GGTATTCCAAATCTTGCCAGTG
	YER006W-DW	GCAATTGGAGCTTGTTTAGTGC
