# Supplementary material for: The SAGA/TREX-2 subunit Sus1 binds widely to transcribed genes and affects mRNA turnover globally
Source: Epigenetics Chromatin. 2018 Mar 29;11:13. doi: 10.1186/s13072-018-0184-2 (PMC5875001; doi:10.1186/s13072-018-0184-2)
Supplement: Supplementary file 7 — Additional file 7. ChIP-exo data analysis. [file 13072_2018_184_MOESM7_ESM.docx]

**Additional Info: ChIP-exo data analysis.**

"Transcription start and end sites (TSS and TES) are from [REF: Xu et al. 2009]. MNase mock and heat shock data is from [REF: Shivaswamy S et al. 2008]"

Xu Z, Wei W, Gagneur J, Perocchi F, Clauder-Munster S, Camblong J, Guffanti E, Stutz F, Huber W, Steinmetz LM. 2009. Bidirectional promoters generate pervasive transcription in yeast. Nature 457: 1033–1037.

Shivaswamy S, Bhinge A, Zhao Y, Jones S, Hirst M, Iyer VR. 2008. Dynamic remodeling of individual nucleosomes across a eukaryotic genome in response to transcriptional perturbation. PLoS Biol 6: e65.
